# The repertoire of testicular extracellular vesicle cargoes and their involvement in inter-compartmental communication associated with spermatogenesis

**DOI:** 10.1186/s12915-022-01268-5

**Published:** 2022-03-29

**Authors:** Kathleen Hoi Kei Choy, Sze Yan Chan, William Lam, Jing Jin, Tingting Zheng, Tin Yu Samuel Law, Sidney Siubun Yu, Weiping Wang, Linxian Li, Gangcai Xie, Howard Chi Ho Yim, Hao Chen, Ellis Kin Lam Fok

**Affiliations:** 1 School of Biomedical Sciences, Faculty of Medicine, The Chinese University of Hong Kong, Hong Kong, SAR China; 2 Dr. Li Dak-Sum Research Centre, University of Hong Kong, Hong Kong, SAR China; 3 Ming Wai Lau Centre for Reparative Medicine, Karolinska Institute, Hong Kong, SAR China; 4 Institute of Reproductive Medicine, Medical School, Nantong University, Nantong, People’s Republic of China; 5 Microbiome Research Centre, St George and Sutherland Clinical School, The University of New South Wales, Sydney, Australia; 6 Sichuan University-The Chinese University of Hong Kong Joint Laboratory for Reproductive Medicine, West China Second University Hospital, Chengdu, People’s Republic of China

**Keywords:** Spermatogenesis, Extracellular vesicles, Testis, Sperm, Male reproduction

## Abstract

**Background:**

Spermatogenesis is regulated by a complex network of intercellular communication processes. Extracellular vesicles (EVs) are one of the important mediators in intercellular communication. Previous reports have demonstrated the involvement of EVs from the epididymis and prostate in sperm maturation and function. However, the presence of EVs in the testis and their potential involvement in spermatogenesis has not been explored. Here, we have established a testis dissociation protocol that allows the isolation and characterization of testicular EVs.

**Results:**

We show that testicular EVs are specifically and efficiently taken up by somatic cells and germ cells, including the spermatozoa in the interstitial space and the seminiferous tubule compartments. We profiled the proteome of testicular EVs and probed the cell types that release them, revealing the potential contributions from the Leydig cells and testicular macrophages. Moreover, we sequenced the small RNA cargoes of testicular EVs and identified sets of small non-coding RNAs that were overlooked in the testis transcriptome. Selected miRNA candidates in testicular EVs were found in sperm RNA payload and demonstrated specific resistance towards ribonuclease A independent of the vesicle membrane. Small molecule inhibition of EV secretion perturbed spermatogenesis via inter-compartmental communication.

**Conclusions:**

Together, our study provides a valuable resource on the repertoire of cargoes carried by testicular EVs and uncovers a physiological function of testicular EVs in inter-compartmental communication associated to spermatogenesis.

**Supplementary Information:**

The online version contains supplementary material available at 10.1186/s12915-022-01268-5.

## Background

Spermatogenesis requires precisely orchestrated cellular communication networks. This involves mechanisms that are ubiquitously used and shared in other organ systems, as well as mechanisms that are unique to the testis [1]. Autocrine and paracrine signaling is a common cell-cell communication in various systems. The testicular microenvironment contains cytokines, peptides, metabolites, hormones, and growth factors involved in stem cell homeostasis, cell differentiation, and cell survival during spermatogenesis [2–9].

Cell junction is another means involved in cell-cell communication. The blood-testis barrier formed by the tight junctions of adjacent Sertoli cells is a unique tight junction required for the compartmentalization of the seminiferous tubules. Other testis-specific cell junctions formed at the Sertoli-Sertoli interface and Sertoli-germ cell interface, also known as the basal ectoplasmic specialization (ES) and apical ES, respectively, are essential for various stages of germ cell development, the phagocytosis of cytoplasmic bodies during spermiogenesis, and the release of sperm to the lumen after the spermiogenesis [8, 10, 11].

Another unique cell-cell communication in the testis is the intracytoplasmic bridges formed in sister spermatogonia after rounds of mitosis. This unique intracellular bridge persists throughout germ cell development and is known to be functionally important [12]. A recent study has demonstrated that the intracytoplasmic bridges can exchange macromolecules such as proteins and RNAs. Besides, the fragmentation of intracytoplasmic bridges may be involved in cell fate determination of the spermatogonial stem cell population [13].

EV is an important mechanism of cell-cell communication. The EVs can be classified into three classes according to their size. The largest size of EVs, typically >1000 nm, is known as the apoptotic bodies. These apoptotic bodies are usually found by blebbing of the apoptotic cells. Microvesicles (MVs) are EVs with sizes ranging from 100 to 1000 nm. They are formed by budding and shedding of the plasma membrane. The smallest size of EVs is exosome, with sizes ranging from 50 to 100 nm. They are produced inside the cells and released via exocytosis [14]. These EVs carry various cargos, including genomic DNA, mRNA, miRNA, and proteins that can be uptake by target cells. Accumulating evidence suggests that proteins on the EVs may be associated with cell recognition and thus target a specific cell type [15]. Furthermore, the delivery of cargoes could regulate the cellular processes of the target cells [16–18], indicating that the EVs are an important and effective way of cell-cell communication.

In the male reproductive tract, EVs from the epididymis and prostate have been characterized. The epididymosomes consisted of two major classes: the CD9+ epididymosomes and ELSPBP1-enriched epididymosomes. The CD9+ epididymosomes transfer their protein cargoes to the spermatozoa and regulate sperm maturation [19]. ELSPBP1-enriched epididymosomes bind preferentially to the dead spermatozoa and quench the reactive oxygen species that may otherwise exert adverse effects on sperm maturation [20]. Besides, epididymosomes also convey RNA to spermatozoa. By comparing the small RNA transcriptome of spermatozoa before and after epididymal maturation, it was shown that the number of miRNAs remains consistent while a marked decrease in piRNA and a significant increase in tRNA fragments (tRFs) is observed after maturation [21–23].

Similar to the epididymosomes, the protein cargoes in prostasomes are also delivered to the spermatozoa and implicate in the survival and motility of sperm via calcium-dependent signaling [24]. Although the prostasome contains DNA, coding and regulatory RNAs with potential modulatory functions, evidence on the transfer of these nucleic acids to the spermatozoa remains scarce.

Surprisingly, although the testis is essential for sperm production, the presence of EVs in the testis and their involvement in spermatogenesis remain largely unknown. Of note, since spermatozoa is transcriptionally inactive, recent studies have postulated that the testicular microenvironment may convey RNA to spermatozoa through EVs [25]. However, the testicular EV is poorly characterized.

Here, we have developed a one-step testis dissociation method for the preparation of single testicular cell suspension. We have isolated and characterized the testicular EVs and showed that the testicular EVs were efficiently taken up by somatic cells and germ cells, including the sperm. We further profiled the protein and small RNA cargos of testicular EVs and probed for the cells that release the testicular EVs. Our study has provided new insights into the intercellular communication in the testis that has broad implications in spermatogenesis.

## Results

### Establishment of one-step testis dissociation method

The isolation of EVs requires the dissociation of the tissue into a single-cell suspension with minimal cell disruption in order to minimize the contamination from membranous organelles. To achieve these, we compared the widely adopted two-step double enzyme digestion protocol, which utilized collagenase and trypsin [26, 27], with a one-step protocol that utilized a non-mammalian, non-bacterial cell dissociation buffer (Additional file 1: Fig. S1A). Single testicular cell suspension can be obtained in both methods with comparable cell yield and cell viability but the one-step method required less hands-on time (Additional file 1: Fig. S1B-D). The one-step testis dissociation protocol also allows the isolation of spermatogonia by magnetic activated cell sorting against Thy1 with a significantly higher yield (Additional file 1: Fig. S1E), suggesting the cell surface antigen was better preserved. To further estimate the cell composition, we analyzed the number of chromosome copies (C) in the testicular single-cell suspension by flow cytometry. The 2C: 4C: 1C ratio in the two-step protocol was 1:2:2 compared to 1:1:8 in the one-step protocol (Additional file 1: Fig. S1F-G). Although the haploid germ cells in the testis also include the secondary spermatocyte, this population was counted 2C. Thus, the increase in 1C must be attributed to the increase in round spermatid, elongated spermatid, and/or spermatozoa. Further sorting of the 1C cells and staining with peanut agglutinin (PNA), a protein used to evaluate the acrosomal development [28], revealed similar percentage of elongated spermatids and a subtle but significant decrease in the percentage of round spermatids in the one-step protocol (Additional file 1: Fig. S1H-I). These results suggest that the one-step method preserves the haploid population, including the spermatozoa that might be lysed in the two-step method and may contribute to the organelle contamination in testicular EVs isolation.

### Testicular EVs contain microvesicles and exosomes

We then isolated the extracellular vesicles from the supernatant of the testicular single-cell suspension by differential centrifugation and commercially available membrane affinity column, two widely adopted methods for EV isolation [29]. Testicular EVs isolated from double-enzymes digestion revealed a plethora of debris surrounding the EVs (Additional file 1: Fig. S2A), suggesting potential contamination of organelles. Consistent with previous reports, differential centrifugation of supernatant from one-step testis dissociation at 10,000 and 100,000 g isolated MVs and exosomes that showed the hallmark cup-shape morphology of EVs, with an average size of 700 and 300 nm, respectively (Additional file 1: Fig. S2B-C). We have also attempted to isolate EVs from conditioned medium obtained from spermatogonia cell lines C18-4 and GC1-spg, and the Sertoli cell line TM4 (Additional file 1: Fig. S2D). Intriguingly, although the starting cell number was comparable between the cell culture (10^8^ cells) and the freshly dissociated testicular cells (10^8^ per mice), the cultured cells released a relatively lower numbers of EVs with variable sizes when examined under the electronic microscope.

**Fig. 1.**
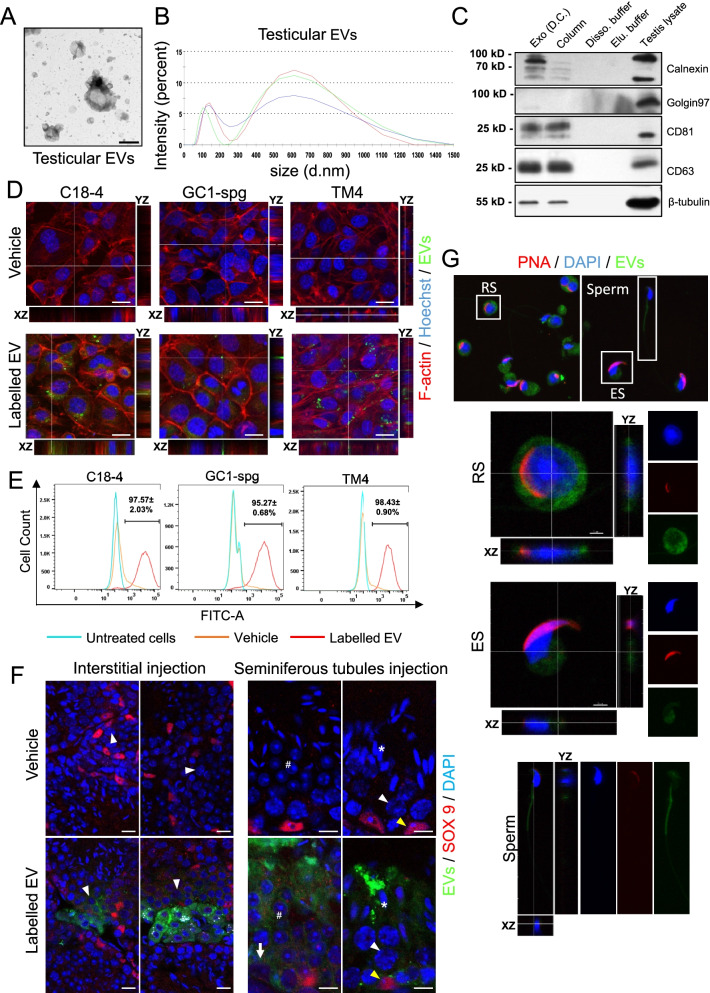
Characterization of testicular extracellular vesicles and their involvement in cell communication in the testicular microenvironment. **A** Representative transmission electron microscopy image of testicular EVs isolated from one-step testis dissociation followed by affinity columns. Scale bar: 500 nm. **B** Size distribution of testicular EVs isolated as in **A** determined by dynamic light scattering. **C** Representative Western blot analysis of EV markers CD81, CD63, and β-tubulin, Golgi marker Golgin 97 and endoplasmic reticulum marker Calnexin in testicular EVs isolated by affinity columns (column) or in exosomes (exo) isolated by differential centrifugation (D.C.). Dissociation buffer and elusion buffer were used as negative control. **D** Representative fluorescent Z-stack images of spermatogonia cell lines (C18-4 and GC1-spg) and Sertoli cell line (TM4) incubated with PKH67 labeled (green) testicular EVs for 24 h. The cells were stained for F-actin (phalloidin, red) and nucleus (Hoechst 33342, blue). Scale bar: 20 μm. **E** Representative histograms showing the percentage of C18-4, GC1-spg, and TM4 cells taken up the testicular EVs as analyzed by flow cytometry. Data is presented as mean ± S.D. **F** Representative fluorescent images showing the uptake of PKH67 labeled testicular EVs in interstitial space (left panel) (*n*=3) and in seminiferous tubules (right panel) (*n*=3). Immunohistochemical staining of Sertoli cell marker SOX9 (red) was shown. Nucleus were counterstained with DAPI (blue). Scale bar: 25 μm. Key: arrow - spermatogonia, arrowhead - spermatocytes, number sign - round spermatids, asterisk - elongated spermatids, and yellow arrowhead - Sertoli cells. **G** Representative fluorescent Z-stack images of isolated round spermatid (RS), elongated spermatid (ES), and testicular sperm incubated with PKH67 (green) labeled testicular EVs for 3h. Nucleus were stained by DAPI (blue), and the acrosomes were stained by PNA (red). Scale bar: 20 μm. Supporting data values available in Additional file 6: Table S5. Uncropped blots available in Additional file 7: Fig S8

In corroboration with the differential centrifugation preparation, testicular EVs isolated by membrane affinity column also revealed two distinct peaks at 600 and 100 nm with similar cup-shaped morphology (Fig. 1A, B). Testicular EVs prepared by both methods expressed EV markers CD81, CD63, and β-tubulin and showed a negligible level of Golgi marker (Fig. 1C). Notably, while the testicular EVs obtained by membrane affinity column showed negligible level of calnexin, an endoplasmic reticulum marker used as a negative control in EV preparation [30], its level was higher in testicular EVs prepared by differential centrifugation (Fig. 1C). We have further compared the EV markers profile in testicular EVs isolated by differential centrifugation of supernatant collected from the two-step double enzyme digestion protocol. The results showed that both the MVs and exosomes isolated by the two-step method expressed lower level of EV markers (Additional file 1: Fig. S2E). These results indicate the presence of exosomes and MVs in testicular EVs. Since relatively pure testicular EVs with minimal organelle contamination can be obtained by membrane affinity column isolation after one-step testis dissociation, we have adopted this protocol in subsequent characterization experiments.

### Uptake of testicular EVs by Sertoli cells and germ cells

EV is an effective way for cell-cell communication. We next sought to investigate the type of cells in the testis that uptake the testicular EVs. We labeled the testicular EVs with PKH fluorescence dye and examined the uptake of fluorescence signals in testicular cell lines and in the mouse testis. Our results showed that treating spermatogonia cell lines C18-4 and GC1-spg and Sertoli cell line TM4 with labeled testicular EV results in the punctated fluorescence signals inside the cells (Fig. 1D). The uptake of labeled testicular EVs in testicular cell lines increased in a time and dose-dependent manner (Additional file 1: Fig. S3). Under optimal uptake conditions, the flow cytometry analysis confirmed more than 95% of cells in each cell line was positive with PKH signals (Fig. 1E), indicating that the tested cell lines were highly efficient in taking up the testicular EVs. Interestingly, compared with the testicular EVs, these cell lines uptake fewer EVs isolated from the conditioned medium of 293 cell line (72% for 293FT-derived EVs vs 95% for testicular EVs) (Additional file 1: Fig. S4), suggesting that efficient uptake was specific to the testicular EVs.

To examine the uptake of testicular EVs in vivo, we have injected the labeled testicular EVs into the interstitial space and the seminiferous tubules. The uptake in various cell types resided in different compartments was investigated by co-staining with Sertoli cell marker SOX9 or germ cell marker TRA98. Our result showed that injection of labeled testicular EVs into the interstitial space results in marked uptake by the interstitial cells and the Leydig cells (Fig. 1F and Additional file 1: Fig. S5). Intriguingly, the fluorescence signals were also observed in the seminiferous tubules at close proximity to the meiotic spermatocytes, suggesting that the testicular EVs were permeable to the basement membrane and the blood-testis barrier and may be involved in inter-compartment communication. Similarly, when the labeled testicular EVs were injected into the seminiferous tubules, a marked uptake was observed in both the Sertoli cells and various stages of germ cells, including the spermatogonia located at the basal compartment (Fig. 1F and Additional file 1: Fig. S5). These results support the notion that the testicular EVs are permeable to the blood-testis barrier, although the possibility that the labeled EVs were uptaken and trafficked inside the Sertoli cells and then delivered to the germ cells across the blood-testis barrier cannot be excluded.

To further investigate the delivery of testicular EVs to testicular germ cells before and after spermiogenesis, we isolated round spermatids, elongated spermatids, and spermatozoa from the seminiferous tubules by mechanical dissociation and treated them with labeled testicular EVs. We observed similar efficient uptake of testicular EVs in the cytoplasm of these cells (Fig. 1G). Together, our results suggest the involvement of testicular EVs in the communication between the Sertoli cells and germ cells, including the spermatozoa.

### Proteome of testicular EVs

**Fig. 2.**
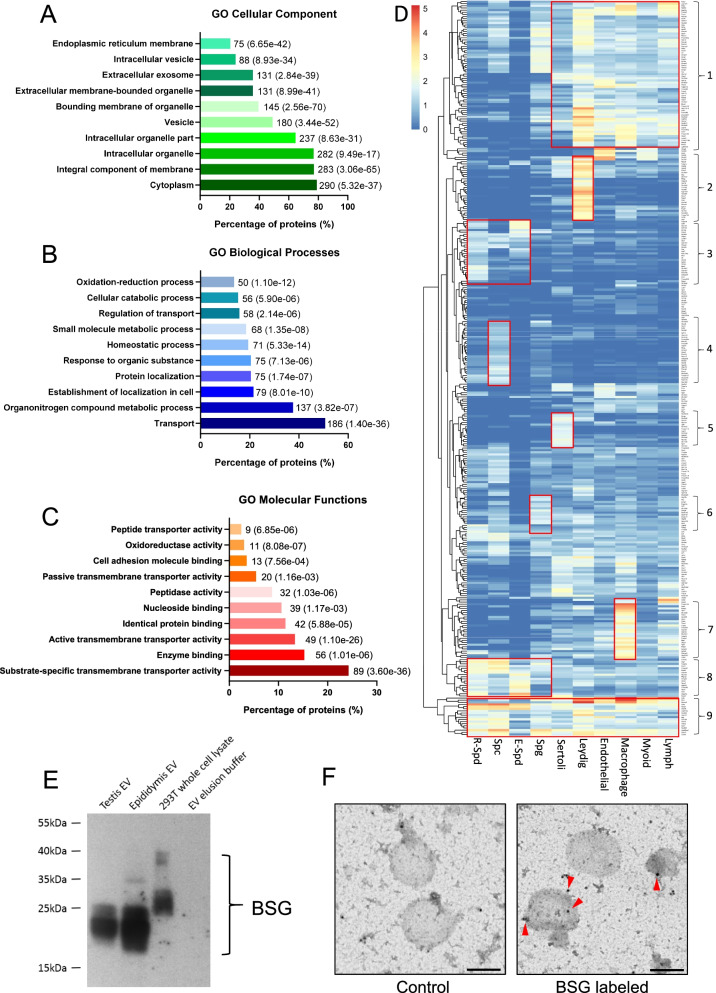
Protein signature of testicular extracellular vesicles. **A**–**C** Gene Ontology analysis on cellular component (**A**), biological process (**B**), and molecular function (**C**) of proteins identified on/in testicular EVs. The number next to the bar indicates the number of genes that fall in the category with corresponding *P* value provided in the blanket. **D** Heat map of genes encoding the proteins identified in the testicular EVs and their corresponding expression in indicated types of testicular cells according to the average expression level of each cell cluster identified by single-cell RNA sequencing analysis of adult mice testes. Cluster 1—somatic cell-enriched; Cluster 2—Leydig cell-enriched; Cluster 3—post-meiotic germ cell-enriched; Cluster 4—spermatocyte-enriched; Cluster 5—Sertoli cell-enriched; Cluster 6—Spermatogonia-enriched; Cluster 7—macrophage-enriched; Cluster 8—germ cell-enriched; Cluster 9—commonly expressed. **E** Western blot analysis of candidate protein Basigin (BSG) identified in testicular EVs. EV isolated from the epididymis was used as a control. **F** Representative immunogold staining images of BSG in testicular EVs. Gold particles are marked by arrowhead. Scale bar: 200 nm. Uncropped blots available in Additional file 7: Fig S8

The protein of EVs may regulate the delivery and modulate the cellular processes in the targeted recipient cells. To profile the proteins carried inside the EVs or on the EV membrane, we examined the testicular EV proteome by liquid chromatography-mass spectrometry (LS-MS/MS). We have identified 553 proteins in two independent EV preparations (Additional file 2: Table S1). Consistent with our previous finding (Fig. 1C), despite the low abundance, EV markers CD63, CD81, and CD9 were identified in the proteomic study. We next mapped the identified proteins to the corresponding genes and carried out a gene ontology (GO) analysis. The results showed that the vesicle, extracellular membrane-bound organelle, and exosome were identified in the GO cellular component analysis (Fig. 2A), suggesting that the testicular EVs shared some degree of similarity in the protein signatures with other systems. Around 50% of the mapped proteins in the testicular EVs were known to be involved in the transport process and 24% possessed substrate-specific transmembrane transporter activity (Fig. 2B, C).

We have also sought to probe for the testicular cell types that release the testicular EVs. To do this, we matched the list of identified proteins with the corresponding gene expression from the single-cell sequencing dataset [31]. We have used the average gene expression level of each cell cluster that cover the majority of expressed genes in a given cell type. We identified 9 clusters of proteins commonly or specifically expressed in various stages of germ cells and various types of somatic cells found in the testicular microenvironment (Fig. 2D and Additional file 3: Table S2), suggesting the secretion of testicular EVs by these cell types. Interestingly, a set of proteins found in the testicular EVs were enriched in the Leydig cells and macrophages (Fig. 2D). An analysis of the mouse phenotype database revealed that among the top 50 proteins with the highest number of unique mapped peptides in the testicular EVs, 11 of them showed a phenotype related to male subfertility or infertility (Additional file 1: Fig. S6), suggesting the importance of these protein cargoes in spermatogenesis. We have validated the proteomic results by Western blot and immunogold labeling of a selected candidate protein Basigin (BSG), a membrane protein/receptor involved in germ cell migration and survival that was also found in the epididymosome [2, 20, 32, 33] (Fig. 2E, F).

### RNA profiles of testicular EVs

**Fig. 3.**
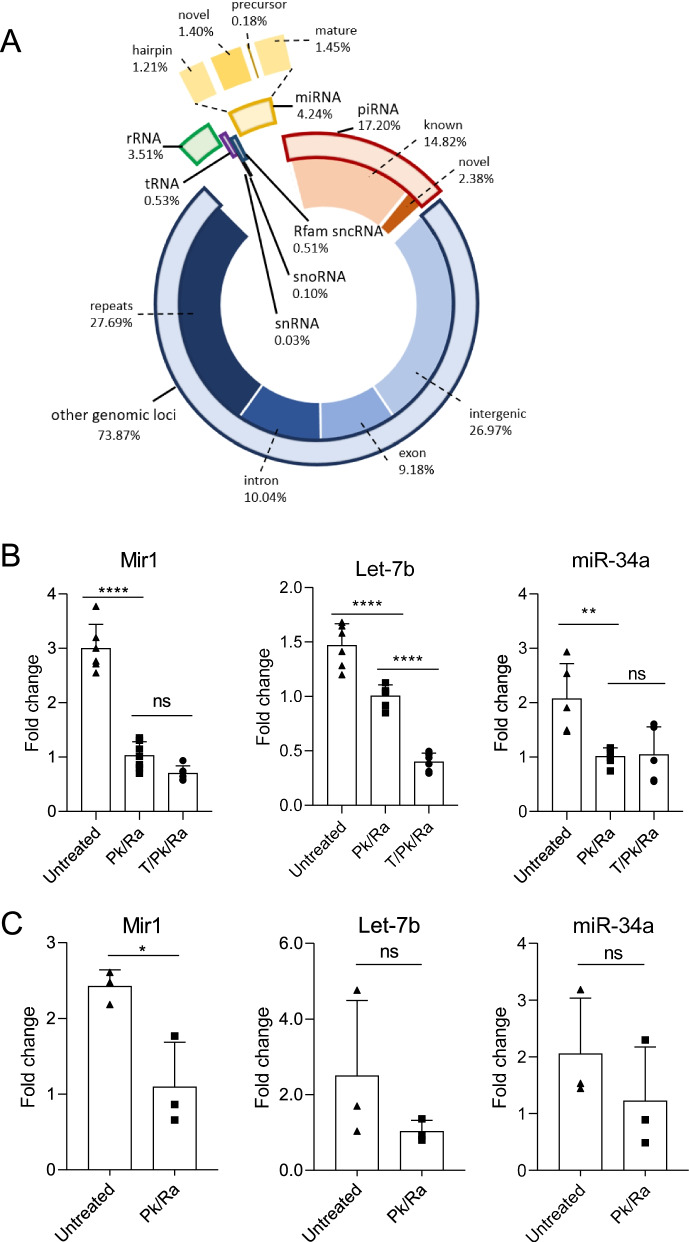
Testicular extracellular vesicles carry miRNA cargoes with differential resistance to RNase in native structure. **A** Diagram showing the distribution of different small RNA species identified in testicular EVs by RNA sequencing and their corresponding percentage counts. Solid bar represents the subclass. Real-time PCR results showing the level of novel Mir1, Let-7b (mature miRNA), and miR-34a (hairpin) in **B** testicular EV samples (*n*=6) and **C** sperm (*n*=3) without treatment (untreated), with proteinase K and RNase A treatment (Pk/Ra) or with Pk/Ra treatment in the presence of 1% TritonX-100 (T). Data is presented as mean ± S.D. **P <0.01, ****P<0.0001, ns—not significant. Supporting data values available in Additional file 6: Table S5

Next, we explored the RNA species carried by the testicular EVs. The RNA was isolated by columns for small RNA after proteinase K and RNase A treatment. Bioanalyzer tracing revealed distinct peaks representing small RNA between 25 and ~200 nucleotide length (Additional file 1: Fig. S7A). Since the small RNA such as miRNA and tRFs have been shown to mediate cell-cell communication and paternal epigenetic inheritance [25, 34], we profiled the small RNA in testicular EVs by small RNA sequencing (Additional file 4: Table S3). Testicular EVs mainly carry small RNA that were mapped to genomic loci that were not annotated for known RNA species (73%). These include reads in repeats, exon, intron, and intergenic regions. The seven RNA subtypes, miRNA, piRNA, rRNA, tRNA, sncRNA, snoRNA, and Rfam sncRNA contributed to the remaining 24% of the total mapped read (Fig. 3A). The piRNA (17%) and miRNA (4%) represent the dominant RNA subtypes among them. Importantly, we have identified sets of novel piRNA and miRNA in the testicular EV samples. These piRNAs and miRNAs may have been overlooked in previous testis transcriptome studies [35, 36], likely because of the low abundance of testicular EV RNA in the total testis RNA.

Unexpectedly, apart from the rRNA and sncRNA that were mapped to the mouse genome, around 10% of rRNA (0.37% of all tags) and 60% of sncRNA (0.3% of all tags) were classified as bacterial rRNA (both large and small subunits) and transfer-messenger RNA (tmRNA) (Additional file 1: Fig. S7B-C), a bacterial RNA molecule with dual tRNA-like and messenger RNA-like properties. This result is consistent with the recent identification of testicular bacteriome in the human testis [37] and the discovery that rRNA and tRNA are released as the dominant RNA species in the bacterial membrane vesicles [38]. Together, these findings suggest the presence of microbial-released vesicles in the testicular EVs.

### RNase resistance of testicular EV miRNA cargos

We then sought to validate the most abundant novel miRNA, Mir1, together with two well-studied miRNAs, miR34a (hairpin), and let-7b (mature). All three miRNAs were identified in testicular EV samples treated with proteinase K and RNase A, suggesting that they were carried inside the EVs and being protected by the EV membrane. The presence of these miRNAs in testicular EVs were also confirmed in the MVs and exosomes isolated from two-step method (Additional file 1: Fig. S7D). Intriguingly, the addition of 1% Triton X-100, which dissolved the EV membrane, together with proteinase K and RNase A treatment significantly reduced the level of let-7b, but not that of Mir1 and miR34a (Fig. 3B), suggesting that these miRNAs possess differential resistance towards RNase A that was not attributed to the EV membrane.

In corroboration with this observation, while RNase A treatment significantly reduced the amount of RNA extracted due to the removal of extracellular cell-free RNA, the presence of Triton X-100 did not further reduce the RNA quantity in testicular EVs (Additional file 1: Fig. S7E), suggesting that the miRNA carried by testicular EVs is resistant to RNase A treatment. Of note, the RNase resistance requires the native RNA structure as alkaline hydrolysis or RNase A treatment after RNA extraction sensitized the candidate miRNAs to RNase A (Additional file 1: Fig. S7F-G), suggesting that the miRNAs in testicular EVs possess specific resistance towards RNases in their native structures. These miRNAs were also carried by spermatozoa (Fig. 3C), suggesting the contribution of testicular EVs to the sperm RNA payload.

### Involvement of testicular EVs in spermatogenesis

**Fig. 4.**
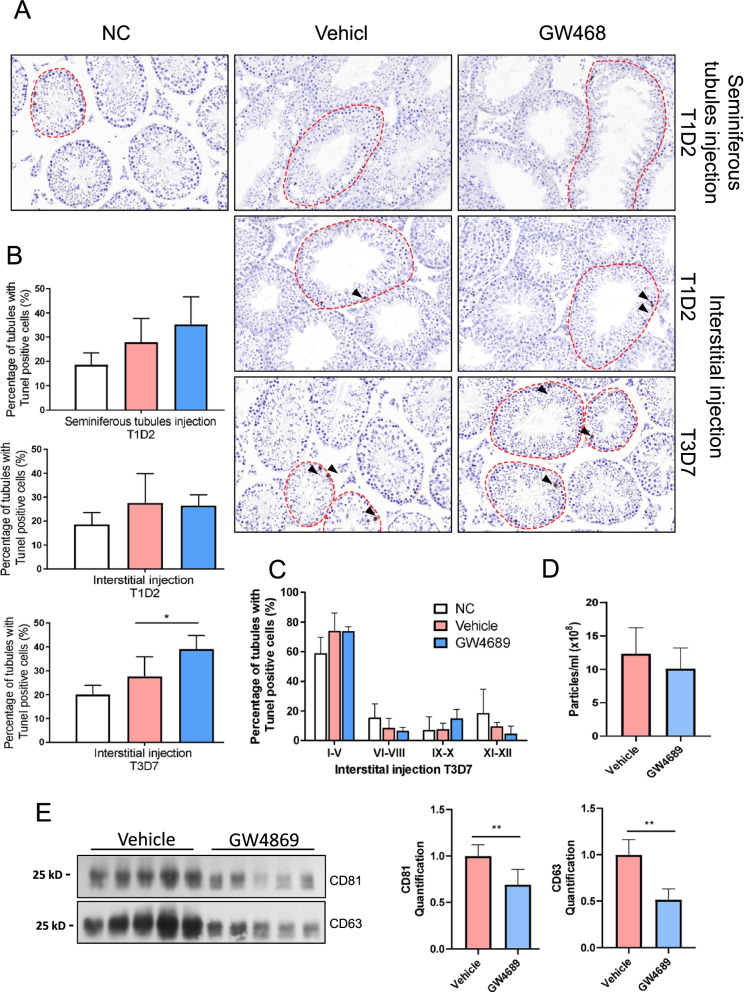
Inhibition of testicular extracellular vesicles elevated apoptosis of germ cells in mice. **A** Representative images of TUNEL assay performed on testes sections treated with GW4869 or vehicle (DMSO) in the seminiferous tubule compartment (upper panel) or interstitium compartment (bottom panels). Single injection (T1) or repeated injections (T3) were performed, and samples were collected 2 days (D2) (*n*=3) or 7 days (D7) (*n*=5) after injection. Arrowheads indicate TUNEL-positive cells, and the dashed lines indicate tubules with TUNEL-positive cells. **B** Quantification from these images is shown. **C** Analysis of proportion of seminiferous tubules with TUNEL-positive cells in different stages of seminiferous tubules. **D** Concentration of testicular EVs isolated from mice treated with GW4869 or vehicle (DMSO) measured by nanoparticle tracking analysis (T3D7). **E** Representative Western blot analysis of EV markers CD63 and CD 81 in equal number of testicular EVs from GW4869 or vehicle (DMSO)-treated mice (T3D7). Quantifications are shown in the bottom panel. Data is presented as mean ± S.D. *P <0.05. Supporting data values available in Additional file 6: Table S5. Uncropped blots available in Additional file 7: Fig S8

Finally, we sought to investigate the involvement of testicular EVs in the development of germ cells. To achieve this, we injected GW4689, a small molecule that inhibits the release of EVs [39], into either the interstitial space or the seminiferous tubules via efferent duct injection. We performed the experiment with two-time points: a single injection followed by a 48-h incubation (T1D2); and three injections (once every 2 days) and a total of 7-day incubation (T3D7). The T3D7 protocol was not feasible for the seminiferous tubule injection. Our results showed that a single injection with a 48-h incubation has no effect on the morphology and germ cell composition as compared to the vehicle control, regardless of the injection sites (Fig. 4A, B). Of note, a repetitive injection in the interstitial space and a longer incubation of 7 days led to a significant increase in the number of seminiferous tubules that showed signs of apoptosis. Intriguingly, apoptosis of Leydig cells and other interstitial cells located at the injection compartment was not observed (Fig. 4A, B). The apoptotic cells were observed across various stages of the seminiferous cycles (Fig. 4C). The decrease in the number of EVs was observed 7 days after repeated injection of GW4689 in the interstitial space (T3D7) (Fig. 4D). Moreover, a significant decrease in the level of CD63 and CD81 was observed in the isolated testicular EVs (Fig. 4E), confirming the effect of repeat injection of GW4689 in inhibiting the exosome secretion. These results suggest that the testicular EVs, at least those in the interstitial space, were involved in normal germ cell development, likely via an inter-compartment communication.

## Discussion

In this study, we have established a one-step tissue dissociation protocol that facilitated the isolation of testicular EVs with limited organelle contamination. Our protocol has omitted the use of trypsin which may disrupt the haploid germ cells/spermatozoa, leading to the release of intracellular membranous content that interferes with the characterization of EVs. Therefore, the proteomic and RNA-sequencing data presented here may represent a comprehensive profile of proteins and small RNAs with a low background interference. Our tissue dissociation protocol also preserves cell surface antigen which allows the isolation of a specific cell type for the primary culture or for antibody-based characterization.

The present study is the first report on the characterization of testicular EVs. We showed that the testis microenvironment consists of microvesicles and exosomes which can be taken up by the somatic cells and germ cells in both the interstitial space and the seminiferous tubules, suggesting that the communication between these cells may involve EVs. Interestingly, our proteomic data reveals two clusters of genes enriched in the Leydig cells and the macrophages, the cells in the interstitial space that uptake the testicular EVs. We postulate that the EVs secreted by the Leydig cells and the macrophages may act locally within the testis and modulate spermatogenesis in the seminiferous tubules. This is further supported by the effects of GW4869 injected into the interstitial space in triggering an increase in the number of apoptotic cells in the seminiferous tubules. Unfortunately, a repetitive injection in the seminiferous tubules was not feasible, and therefore, the effects of testicular EVs within the seminiferous tubules cannot be investigated in the current study.

Besides, our result showed the presence of a non-negligible amount of microbial EVs and their RNA cargoes in the testicular microenvironment. While the function of the bacterial rRNA and tmRNA in testicular microbial EVs remain elusive, accumulating evidence has suggested a microbe-host communication where the small RNA in microbial EVs is internalized by the host cells and regulate cytokine production, signaling pathways, and miRNA-like translational regulation [40, 41]. In fact, microbial RNA is present in sperm RNA and has been postulated to reflect the diversity of the associated bacteriome [42]. These previous results and our findings collectively support the hypothesis that the sperm can internalize microbial EVs and carry the small RNA cargoes within. The involvement of these microbial EV small RNAs in the regulation of spermatogenesis and in the epigenetic inheritance of microbiome warrant future investigation.

Our proteomic study revealed a plethora of proteins in the testicular EVs that have been implicated in male fertility, suggesting that the communication mediated by testicular EVs is required for spermatogenesis. Indeed, mouse genome informatic databases show that chemically induced mutation in the *Smpd3* gene, which encodes for an enzyme required for EV release, reduced fertility [43]. Also, gene knockout of Smpd3 shows delayed testis development and perturbs spermatogenesis at secondary spermatocytes [44]. These results support an important role of testicular EVs in spermatogenesis. Surprisingly, although the Sertoli cell line and spermatogonial cell lines are capable of taking up the EVs, these cells did not release a measurable quantity of EVs in the culture condition tested. It is known that the efficiency of in vitro spermatogenesis is low. It is plausible that the absence of EV-mediated cell communication underlies the poor differentiation efficiency of germ cells in vitro.

Our results showed that testicular cells, including spermatogonia, Sertoli cells, and spermatozoa, are highly efficient in taking up testicular EVs but not EVs released by other somatic cells. This suggests the involvement of a specific targeting mechanism apart from the direct fusion of the membrane [23]. Notably, around 10% of the proteins identified in the testicular EVs are involved in protein binding, suggesting a potential receptor-mediated uptake. Interestingly, our results also showed that testicular spermatozoa are highly efficient in taking up the testicular EVs which may be a route to convey RNA into the transcriptionally inert spermatozoa.

EVs are known to carry RNA cargoes that are protected from RNase by the lipid membrane. As expected, the candidate miRNAs in the testicular EVs were protected from RNase A. A portion of the miRNAs demonstrated another level of RNase resistance that requires a native conformation independent of RNA-binding proteins. This could be attributed to a unique vesicle membrane architecture that is more resistant to detergent treatment and thus the incomplete digestion of the membrane or an undefined RNA protection mechanism. The physiological significance of this potential RNase resistance warrants further investigation. Since the testicular EVs can deliver the cargoes to the spermatozoa, we postulate that such stable miRNAs may be inheritable via the sperm epigenome and mediates paternal inheritance. Further, the mechanistic study on the RNase resistance of miRNAs may shed light on the synthesis of stable RNA molecules for gene therapy and genetic engineering.

### Limitations of the study

While this study is the first to report the involvement of the extracellular vesicles in spermatogenesis in vivo, there are a number of caveats that warrant further investigation and the current results should be interpreted with caution.

First, the injection of GW4689 in the interstitial compartment results in the global inhibition of exosome secretion without targeting a specific population of exosomes or a specific cell type that releases them. Therefore, the donor and the recipient of the exosome-mediated intercellular communication remain undefined. Besides, since the cell body of Sertoli cells encompasses both the basal and adluminal compartment, the possibility of GW4689 injected in the interstitial space to reach the Sertoli cells and exert the effects in the seminiferous tubules cannot be excluded.

Secondly, while we observed the decrease in EV markers CD63 and CD81 after GW4689 treatment, the proteomic characterization of testicular EVs revealed a unique protein signature where these EV markers represent a small population of unique mapped peptides. In this case, the effect of GW4698 on the release of testicular EVs may have been overestimated, and the absence of effect after GW4689 treatment in the seminiferous tubule injection model should not be over-interpreted.

Lastly and importantly, the increase in the percentage of seminiferous tubules with signs of apoptosis after repeated GW4689 treatment (T3D7) in the interstitial compartment suggested a perturbation in spermatogenesis. Nonetheless, since apoptosis is a physiological event during spermatogenesis, particularly during meiosis, the observed effect could be attributed to an increase in apoptosis of germ cells or an arrest in a specific stage of the seminiferous tubules where physiological apoptosis is more readily observed. Further investigation is required to characterize the effect of inhibiting exosome secretion on spermatogenesis.

## Conclusions

Taken together, our study has successfully isolated the testicular EVs and characterized the protein and small RNA cargoes, providing valuable resources for future studies. We have further demonstrated its potential involvement in testicular cell communication and revealed a unique protection mechanism of the small RNA cargoes from RNases degradation, which may be involved in spermatogenesis.

## Methods

### Animals

All animal experiments were approved by the Animal Research Ethics Committee of the Chinese University of Hong Kong (20-081-ECS). Eight-week-old male C57BL/6 mice were purchased from the laboratory animal service center (LASEC) of the Chinese University of Hong Kong.

### Testis dissociation

The mice were sacrificed by cervical dislocation. For the one-step method, seminiferous tubules were suspended in the Accumax Cell Aggregate Dissociation Medium (20mg/ml) (Invitrogen; 00-4666-56) and incubated on a rocking platform at room temperature for 1 h. The dispersed cells were filtered through a 40-um-cell strainer (Corning; 352340) to a 50-ml falcon tube and washed with PBS. Filtered cells were pelleted at 300g for 5 min, and the EV-containing supernatant was further filtered through using a 0.8-um syringe filter (Millipore; SLAA033SB).

The two-step double-enzyme digest method was performed as described [26], and seminiferous tubules were suspended in PBS containing collagenase (1 mg/ml) (Sigma-Aldrich) and incubated at 37°C for 10 min. The cells were washed once with PBS and further incubated in Trypsin (0.25%) (Gibco) and Dnase I (5mg/ml) (Sigma-Aldrich) at 37°C for 10 min. Ten percent of FBS were added to inactivate the trypsin upon single-cell suspension, and the dispersed cells were washed with PBS and Dnase I before filtered through using a 40-um cell strainer. Filtered cells were pelleted at 300g for 5 min, and all EV-containing supernatants were collected and filtered through using a 0.8-um syringe filter.

### Isolation of EVs

For the affinity column method, EV was isolated using exoEasy Maxi Kit (Qiagen, 76064) according to the manufacturer’s instructions and further concentrated by Ultracentrifugation (Hitachi CS-150GXII Micro UITRAcentrifuge), performed at 100,000g for 90 min at 4°C.

For the differential centrifugation method, MVs were isolated by centrifugation, at 10,000g for 30 min at 4°C using Beckman Avanti J-E Centrifuge. Exosomes were then isolated by centrifugation at 100,000g for 90 min at 4°C using Beckman Coulter Optima XPN-100 ultracentrifuge.

### Transmission electron microscope

Morphology of testicular EVs was observed using a transmission electron microscope. EVs from two testes were resuspended in 20 μl PBS and fixed with 2% paraformaldehyde until use. Ten microliters of EVs were added onto the formvar grid (200 mesh) for 30–60 min, and excess fluid was removed with filter paper. EVs were fixed with 1% glutaraldehyde for 10 min, followed by negative staining with 2% uranyl acetate for 2 min, and images were captured using a Hitachi H-7700 transmission electron microscope.

For immunogold labeling, fixed EVs were applied to the formvar grids (200 mesh), after washing with PBS/50mM glycine to quench-free aldehyde groups for 3 min for a total of four washes, grids were blocked with PBS/5% BSA for 1 h, followed by primary antibody to Basigin (Abcam; ab188190, 1:20) in PBS/1% BSA and gold labelled secondary antibody (Boster; GA1013, 1:20) in PBS/1% BSA incubation for 1h. The grids were then stained with 2% uranyl acetate, and images were captured as described above.

### Dynamic light scattering

Concentrated EVs were resuspended in 100 μl PBS, and 50 μl of EVs was diluted in 950 μl PBS for determination of size distribution using the dynamic light scattering (Zetasizer Nano-ZS system). Three independent measurements were performed for each sample.

### Nanoparticle tracking analysis

The concentration of testicular EVs was measured by the Nanosight LM14C (Malvern) instrument. The samples were diluted with filtered PBS to obtain the optimal measurement condition of 5–15×10^8^ particles/ml. Thirty second videos were recorded of each sample, and the concentration of testicular EVs was analyzed and calculated with nanosight software using the Stokes-Einstein equation.

### Western blot

Protein lysate from EVs or cells was obtained by incubating with RIPA buffer with protease inhibitors. A total of 40 μg protein were electrophoresed under denaturing conditions on 12% polyacrylamide gels and transferred onto the PVDF membranes. After blocking with 5% non-fat milk for 1 h, the membranes were immunoblotted with primary antibodies to CD 81 (Santa Cruz; sc-166029, 1:1000), CD 63 (Santa Cruz; sc-365604, 1:1000), β-tubulin (Cell Signaling; 2146, 1:2000), Calnexin (Immnoway; YT0613, 1:1000), Golgin 97 (Santa Cruz; sc-59820, 1:1000), and BSG (Abcam; ab188190, 1:1000) overnight at 4 °C, followed by the relevant HRP-conjugated secondary antibodies incubation for 1 h at room temperature. Bands were visualized by Prime ECL.

### Cell line

The cell lines used in this study were C18-4 mouse undifferentiated spermatogonia cell line, GC1-spg mouse spermatogonia cell line, and TM4 mouse Sertoli cell line. C18-4 cells were maintained in DMEM medium supplemented with 10% FBS, 1mM sodium pyruvate, 1% l-glutamine, and 1 x nonessential amino acids at 35 °C with 5% CO_2_. GC1-spg cells and TM4 cells were cultured in DMEM or DMEM/F12 medium respectively containing 10% FBS at 37 °C with 5% CO_2_. C18-4, GC1-spg, and TM4 cells were seeded on five 15-mm dishes containing 20 ml of cell medium with exosome-depleted FBS (Thermo Fisher, A2720803), and cell medium was collected for EV isolation when cells were grown to 95% confluency.

### Uptake of EVs

Concentrated EVs were labeled with PKH67 Green Fluorescent Cell Linker Mini Kit (Sigma-Aldrich, MINI67-1KT) according to the manufacturer’s instructions. EV concentrations were determined by protein assay. To study the EV uptake in testicular cells or somatic cells in vitro, indicated amount of labeled EVs or vehicle controls were incubated with C18-4, TM4, and GC1-spg cell line when cells reached 60–70% of confluency in 24-well plates for indicated time-points. The uptake of EVs in cells or spermatozoa was analyzed by immunofluorescence staining or flow cytometry.

To study the EV uptake in the mouse testis, mice were fully anesthetized with 100mg/kg ketamine and 10 mg/kg xylazine mix and labeled EVs or vehicle controls (PKH67 dye) were injected to the seminiferous tubules via efferent duct or interstitium of the mouse testes [45–47]. After 24 h, mice were sacrificed and the testes were collected. Snap-frozen testicular sections (5 μm) were fixed with 4% paraformaldehyde for 15 min, and slides were mounted in an antifade mounting medium with DAPI.

For the study of EV uptakes in primary testicular haploid germ cells, the haploid germ cells close to the lumen of the seminiferous tubules were isolated by manual pipetting of the seminiferous tubules followed by a Percoll gradient purification. Briefly, the seminiferous tubules were spread and rocked (~80 rpm) in 1 x HBSS at a volume of 1 ml per 20 mg tissue at room temperature for 1 h. The cells were dispersed by pipetting 20 times once every 30 min. Then, the cell suspension was filtered through a 40-μm cell strainer and pelleted by spinning at 500 x *g* for 5 min. The primary germ cells were purified by 22.5–45% (w/v 1 x HBSS) Percoll gradients. The 22.5% percoll gradient layer was obtained, and 15 mL of 1 x PBS was added followed by centrifugation at 500 x *g* for 5 min to remove the residual Percoll solution. The primary germ cells enriched in haploid population cell pellets were counted, and 1 x 10^6^ cells were used for tEV treatment. Staging of the haploid germ cells was performed by PNA staining after the tEV uptake (see below).

### Thy1+ isolation with MACS

1x10^7^ cells from one-step and two-step double-enzyme digest methods were resuspended in 200ul 2% FBS DMEM and incubated with 20ul of Biotinylated Thy1.2 CD90.2 primary antibody (BD IMag™, 551518) on ice for 15 min on a slow-rocking platform. Cells were washed with PBS and resuspended with 500ul 2% FBS DMEM before loaded into the pre-calibrated MS column (Miltenyi Biotec, 130-042-201) to allow Thy1^-^ cells to flow through by gravity. Columns were washed twice with 500ul 2% FBS DMEM, and finally, Thy1+ cells were eluted with 1ml 2% FBS DMEM.

### Flow cytometry

Cells collected from one-step and two-step double-enzyme digest methods were washed once with PBS and permeabilized overnight with 70% ethanol at −20°C. Samples were stained with 40ug/ml PI in PBS and 100ug/ml Rnase A and incubated at 37°C for 30 min in dark. PI-stained cells were analyzed with BD LSR Cell Analyzer, counting 10,000–20,000 events per sample.

Flow cytometry was used to determine the percentage of EV uptake in cell lines. After the incubation period with labeled EVs or vehicle control, cells were washed with PBS once and trypsinized, pelleted by centrifugation at 1000 rpm for 5 min, and resuspended in 300 μl of cell medium. Fluorescence-positive cells were analyzed BD LSR Fortessa Cell Analyzer, counting 50,000 cells for each sample.

### Immunofluorescence staining

Cell lines or primary testicular germ cells were fixed in 4% paraformaldehyde for 15 min, permeabilized with 0.1% Triton X-100 for 10 min followed by incubation with Alexa Fluor 568 Phalloidin (dilution 1:200) for 20 min or Lectin PNA (Invitrogen; L32458, 1 μg/ml) for 1 h. Cells were counterstained with Hoechst 33342 (1:2000) or DAPI for 10 min. The fluorescence images were captured with Leica TCS SP8 confocal microscope, and Z-stack images were analyzed with Imaris Cell Imaging Software.

### Alkali/detergent and nuclease treatment of testis EV

Prior to RNA extraction, EV/sperm samples were treated with or without 1% Triton X-100 (Boehringer Mannheim, 1332481) and incubated at room temperature for 30 min to disrupt the membrane. The samples were then treated with 0.05ug/ul Proteinase K (Qiagen, 1014023) at 37°C for 10 min, inactivated with 5-mM PMSF (Sigma-Aldrich, P7627) at room temperature for 10 min and then treated with 0.5ug/ul Rnase A (Thermo Scientific™, EN0531) at 37°C for 20 min. Alkaline hydrolysis was performed as described [48]. Briefly, the samples were first incubated at 65°C for 1 h in 0.1M Tris (pH 8.0) and 1M NaOH, then neutralized with 1M HCl at a ratio of 2.33:1:1.

### RNA extraction, reverse transcription and real-time PCR

The total RNA was extracted using the miRNeasy Mini Kit (Qiagen, 217004) with RNase-Free DNase Set (Qiagen, 79254) according to the manufacturer’s instructions. A 10pg of mirVana miRNA mimic miR-199a-5p (ThermoFisher, Assay ID MC10893 and 002304) was added to 0.1–0.5ug of the total RNA as a spike-in control for RT-qPCR normalization. Reverse transcription was performed using PrimeScript™ RT Master Mix (TaKaRa, RR036A) for novel miRNA and TaqMan™ MicroRNA Reverse Transcription Kit (Applied Biosystems, 4366597) for the candidate miRNAs let-7b-5p (ThermoFisher, Assay ID 000378) and miR-34a-5p (ThermoFisher, Assay ID 000426). One microliter of cDNA was used for a real-time PCR using TaqMan™ Universal PCR Master Mix (Applied Biosystems, 4364340) on the ABI QuantStudio 7 Flex Real-Time RCR system. Primers and probes used are listed in the Additional file 5: Table S4. Results were calculated with 2(−ΔΔCt).

### Small RNA sequencing

EV RNA after treatment with RNase A and proteinase K was extracted as described above and used for small RNA-Seq which was performed by BGI (Shenzhen, China). Small-RNA libraries were prepared, and the PCR products were sequenced using BGISEQ-500 technology. After elimination of low-quality reads, clean reads were mapped to reference genome and to other sRNA databases using Bowtie2 and cmsearch [49, 50]. Classification of sRNA follow the priority rule: MiRbase> pirnabank> snoRNA(human/plant)> Rfam> other sRNA to ensure unique map of each entry. Novel miRNAs and piRNAs were predicted using miRDeep2 and Piano, respectively [51, 52].

### LC-MS/MS

Testicular EV proteins were extracted with RIPA buffer, and LC-MS/MS proteomics analyses were performed by Winninnovate Bio (Shenzhen, China). Gene ontology (GO) enrichment and KEGG pathway enrichment were analyzed. To map, the cell types that express the identified proteins, we analysed the expression of genes encoding the identified proteins by taking the average expression level of these genes in different cell clusters from single cell RNA sequencing analysis of adult mouse testes [31].

### GW4869 Treatment

To study the effect of exosome generation inhibitor GW4869 (Sigma-Aldrich, D1692) on spermatogenesis in mouse, GW4869 dissolved in DMSO (20 μM, 20μl/testis) or vehicle (DMSO) were injected into the mice seminiferous tubules or interstitium of testes as described above. Treatments were conducted once or every other day for three times. After the indicated treatment time, mice were sacrificed and testes were fixed in 4% paraformaldehyde, dehydrated, and embedded in paraffin. The TUNEL assay was performed on the testis sections (5 μm) with ApopTag plus peroxidase in situ apoptosis detection kit (Millipore, S7101). The total number of seminiferous tubules and the number of tubules with TUNEL-positive cells were counted in at least 10 random fields of the testis sections. The percentage of tubules with positive TUNEL cells were then calculated.

### Statistical analysis

Statistical analysis was carried out using GraphPad Prism version 8.02. Student’s *t* test was used to compare group means. Ordinary one-way analysis of variance (ANOVA) with Dunnett’s post hoc test was used for analysis involving three or more groups of samples. A *P* value of <0.05 was considered significant.

## Supplementary Information

**Additional file 1: Figure S1.** Comparison of one-step and double-enzyme digestion methods for mouse testis dissociation. **Figure S2.** Isolation and characterization of extracellular vesicles in mouse testis and testicular cell lines. **Figure S3.** Time and dose-dependent uptake of testicular extracellular vesicles by testicular cell lines. **Figure S4.** Uptake of extracellular vesicles secreted from 293FT cells by C18-4 cells. **Figure S5.** Uptake of testicular EVs in testicular cells of interstitial and seminiferous tubules compartments. **Figure S6.** Phenotypes of male mice with knockout of genes encoding the proteins identified in testicular EVs. **Figure S7.** Characterization of the small RNA cargoes of testicular EVs.

**Additional file 2: Table S1.** List of proteins identified in testicular EVs.

**Additional file 3: Table S2.** Expression profiles of genes encoding for the proteins identified in testicular EVs.

**Additional file 4: Table S3.** List of small RNAs identified in testicular EVs.

**Additional file 5: Table S4.** Oligo information.

**Additional file 6: Table S5.** Individual data values. Individual data values available of experiments performed where n<6.

**Additional file 7: Figure S8.** Uncropped blots.

## Data Availability

All data generated or analyzed during this study are included in this published article, its supplementary information files and publicly available repositories: Proteomic data is available on the PRIDE database (https://www.ebi.ac.uk/pride/; Project accession: PXD021766). Small RNA sequencing data is available on the GEO database (https://www.ncbi.nlm.nih.gov/geo/; Accession number: GSE158402). Individual data values for experiment with *n*<6 are provided in Additional file 6: Table S5. Original uncropped blots are available in Additional file 7: Fig S8).

## Abbreviations

BSA: Bovine serum albumin
BSG: Basigin
DAPI: 4',6-Diamidino-2-phenylindole
DMEM: Dulbecco’s modified Eagle medium
DMSO: Dimethyl sulfoxide
DNase: Deoxyribonuclease
ECL: Enhanced chemiluminescence
ES: Ectoplasmic specialization
EVs: Extracellular vesicles
FBS: Fetal bovine serum
GO: Gene ontology
KEGG: Kyoto Encyclopedia of Genes and Genomes
LASEC: Laboratory animal service center
LS-MS/MS: Liquid chromatography-mass spectrometry
miRNA: MicroRNA
MVs: Microvesicles
PI: Propidium iodide
piRNA: Piwi-interacting RNA
PMSF: Phenylmethylsulfonyl fluoride
PNA: Peanut agglutinin
RNase: Ribonuclease
rRNA: Ribosomal RNA
sncRNA: Small non-coding RNA
snoRNA: Small nucleolar RNA
tmRNA: Transfer-messenger RNA
tRFs: tRNA fragments
tRNA: Transfer RNA
TUNEL: Terminal deoxynucleotidyl transferase dUTP nick-end labeling
