# Supplementary material for: The repertoire of testicular extracellular vesicle cargoes and their involvement in inter-compartmental communication associated with spermatogenesis
Source: BMC Biol. 2022 Mar 29;20:78. doi: 10.1186/s12915-022-01268-5 (PMC8966158; doi:10.1186/s12915-022-01268-5)
Supplement: Supplementary file 1 — Additional file 1: Figure S1. Comparison of one-step and double-enzyme digestion methods for mouse testis dissociation. Figure S2. Isolation and characterization of extracellular vesicles in mouse testis and testicular cell lines. Figure S3. Time and dose-dependent uptake of testicular extracellular vesicles by testicular cell lines. Figure S4. Uptake of extracellular vesicles secreted from 293FT cells by C18-4 cells. Figure S5. Uptake of testicular EVs in testicular cells of interstitial and seminiferous tubules compartments. Figure S6. Phenotypes of male mice with knockout of genes encoding the proteins identified in testicular EVs. Figure S7. Characterization of the small RNA cargoes of testicular EVs. [file 12915_2022_1268_MOESM1_ESM.pdf]

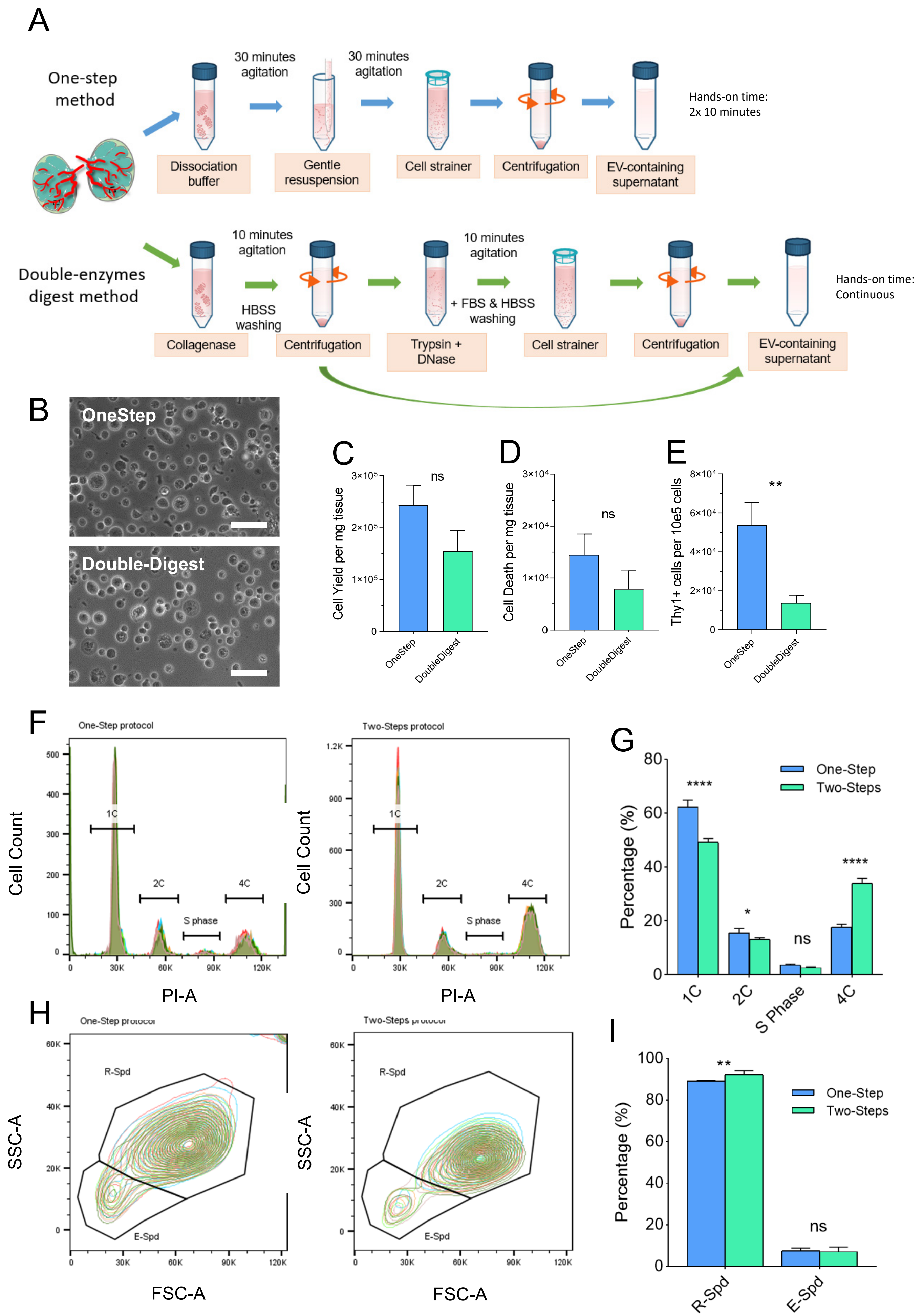

Additional File 1: Fig. S1| Comparison of one-step and double-enzyme digestion methods for mouse testis dissociation.

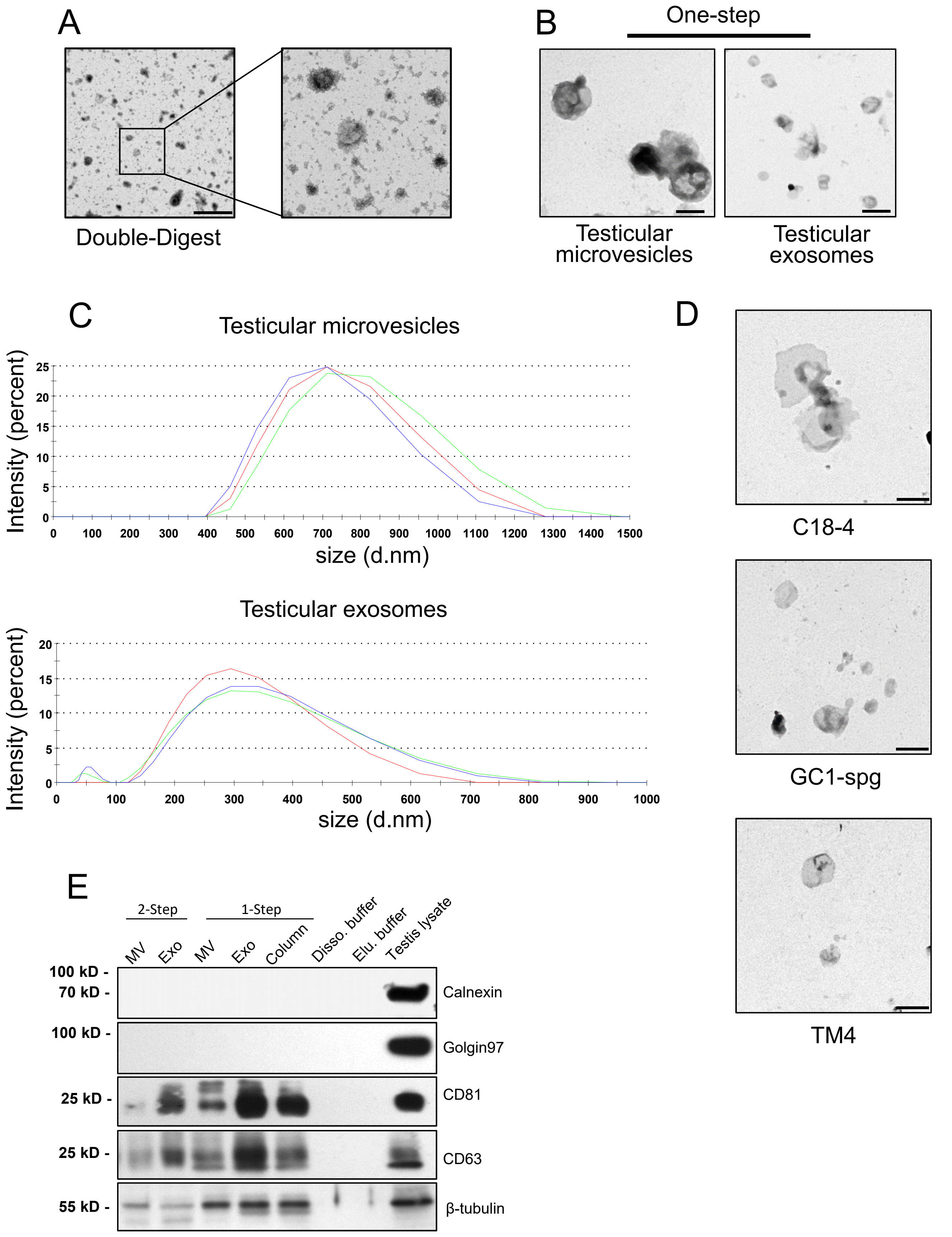

Additional File 1: Fig. S2| Isolation and characterization of extracellular vesicles in mouse testes and testicular cell lines

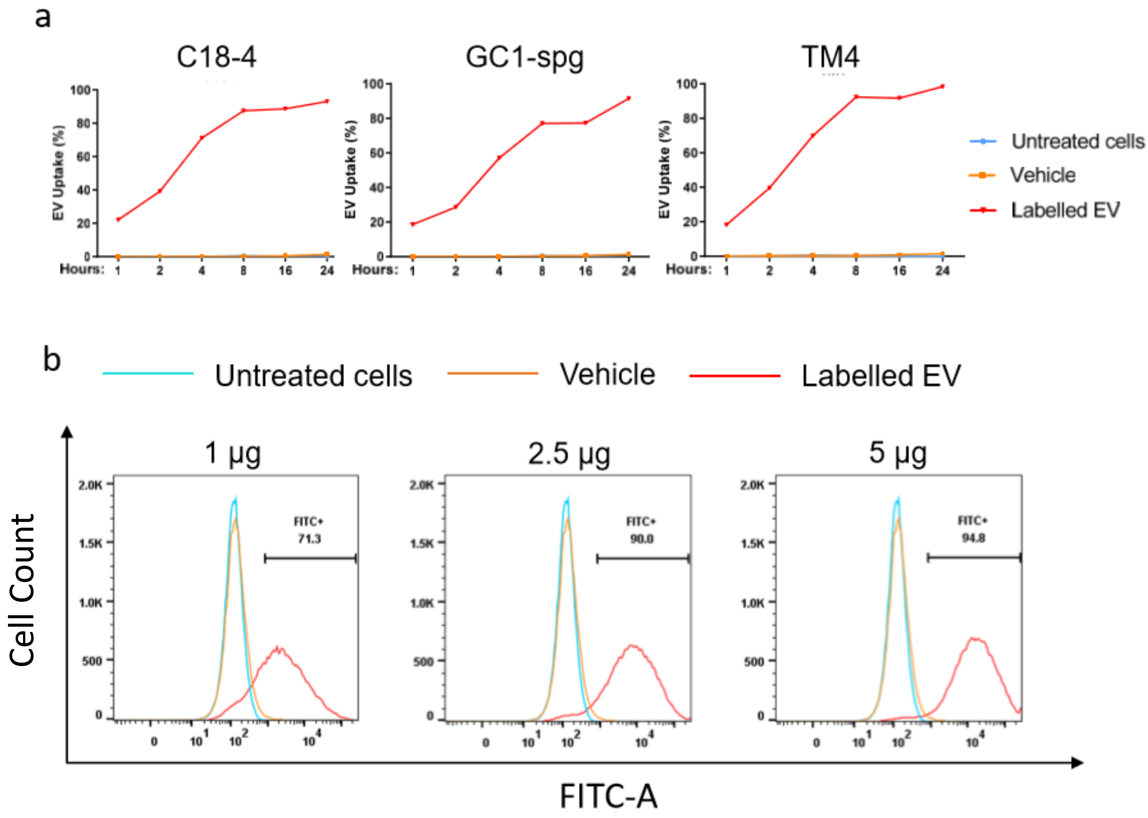

Additional file 1: Fig. S3| Time and dose-dependent uptake of testicular extracellular vesicles by testicular cell lines.

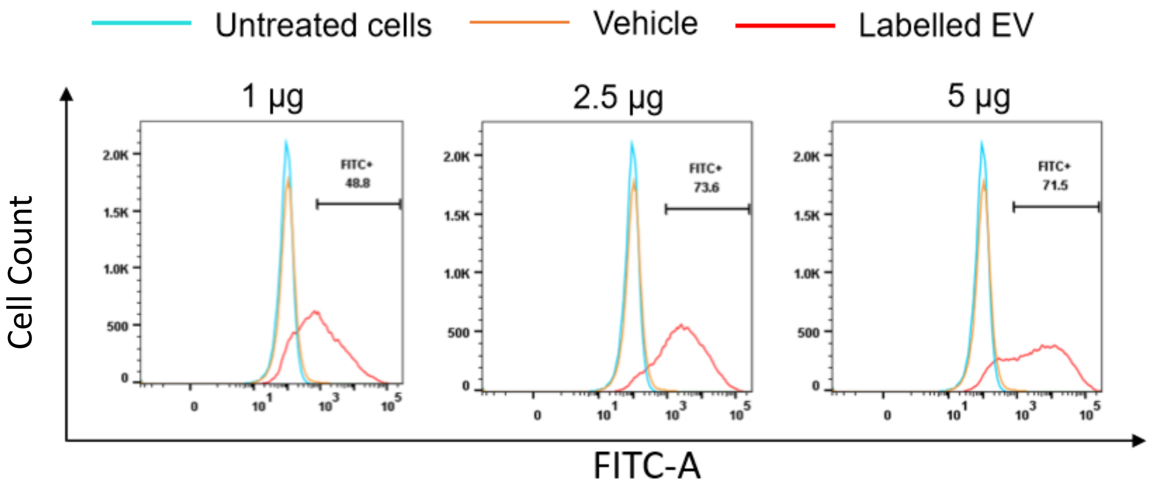

Additional file 1: Fig. S4| Uptake of extracellular vesicles secreted from 293FT cells by C18-4 cells.

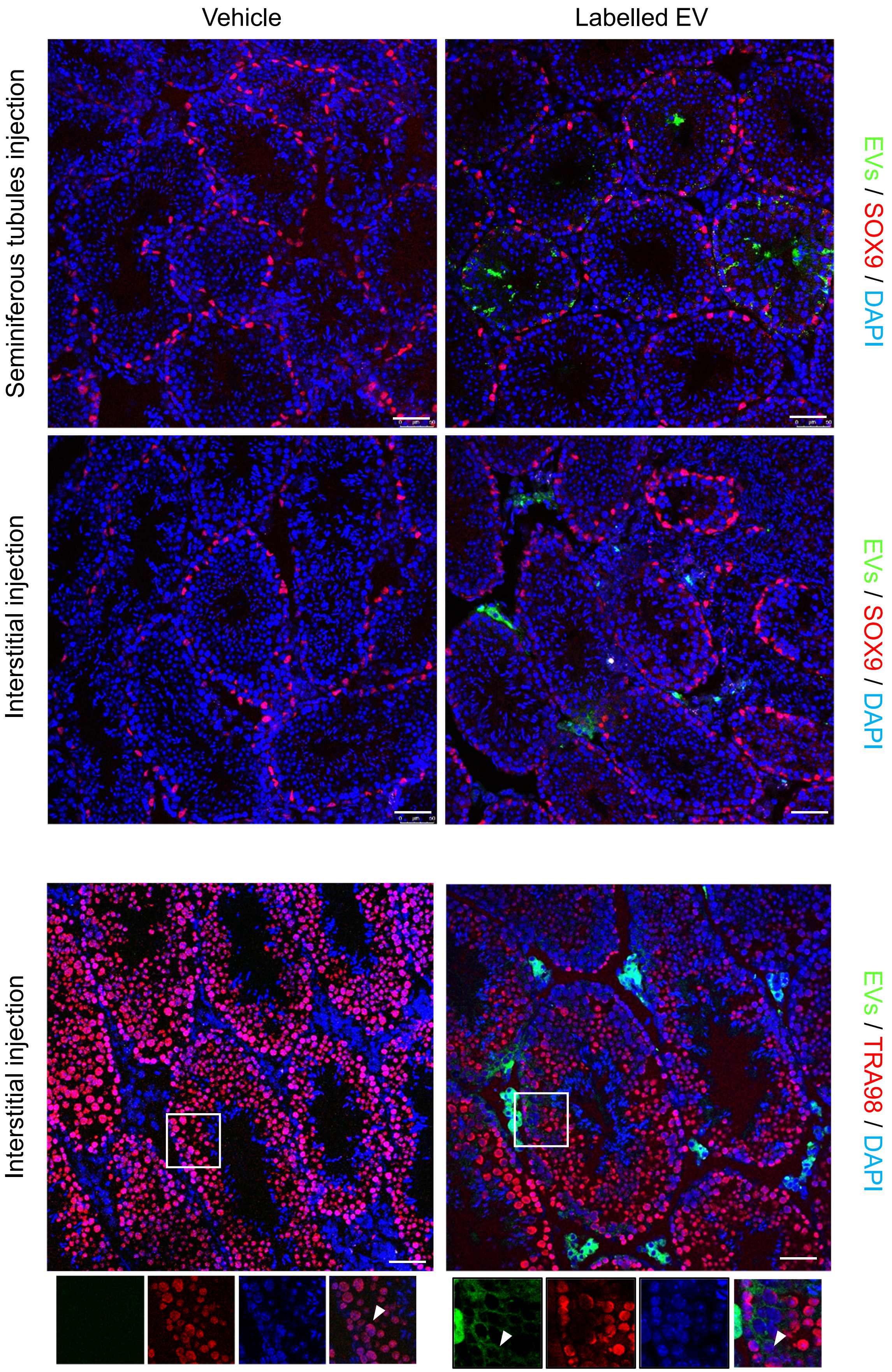

Additional file 1: Fig. S5| Uptake of testicular EVs in testicular cells of interstitial and seminiferous tubules compartments.

Gene name	Accession	Protein Name	Phenotype information
Slc2a3	P32037	Solute carrier family 2, facilitated glucose transporter member 3	Decrease litter size
Vdac3	Q60931	Voltage-dependent anion-selective channel protein 3	Male infertility
Gpx4	F7A8H6	Glutathione peroxidase	Male infertility
Slc25a3	Q8VEM8	Phosphate carrier protein mitochondrial	Testis atrophy
Ace	P09470	Angiotensin-converting enzyme	Reduced male fertility
Sypl1	O09117	Synaptophysin-like protein 1	Male infertility
Gapdh	A0A0R4J0X7	Glyceraldehyde-3-phosphate dehydrogenase	Male infertility
Atp2a2	O55143	Sarcoplasmic/endoplasmic reticulum calcium ATPase 2	Decrease little size
Aqp7	O54794	Aquaporin-7	Abnormal sperm
Bsg	P18572	Basigin	Male infertility
Snrpe	P62305	Small nuclear ribonucleoprotein E	Testis atrophy

Additional file 1: Fig. S6| Phenotype of male mice with knockout of genes encoding top proteins identified in testicular EVs.

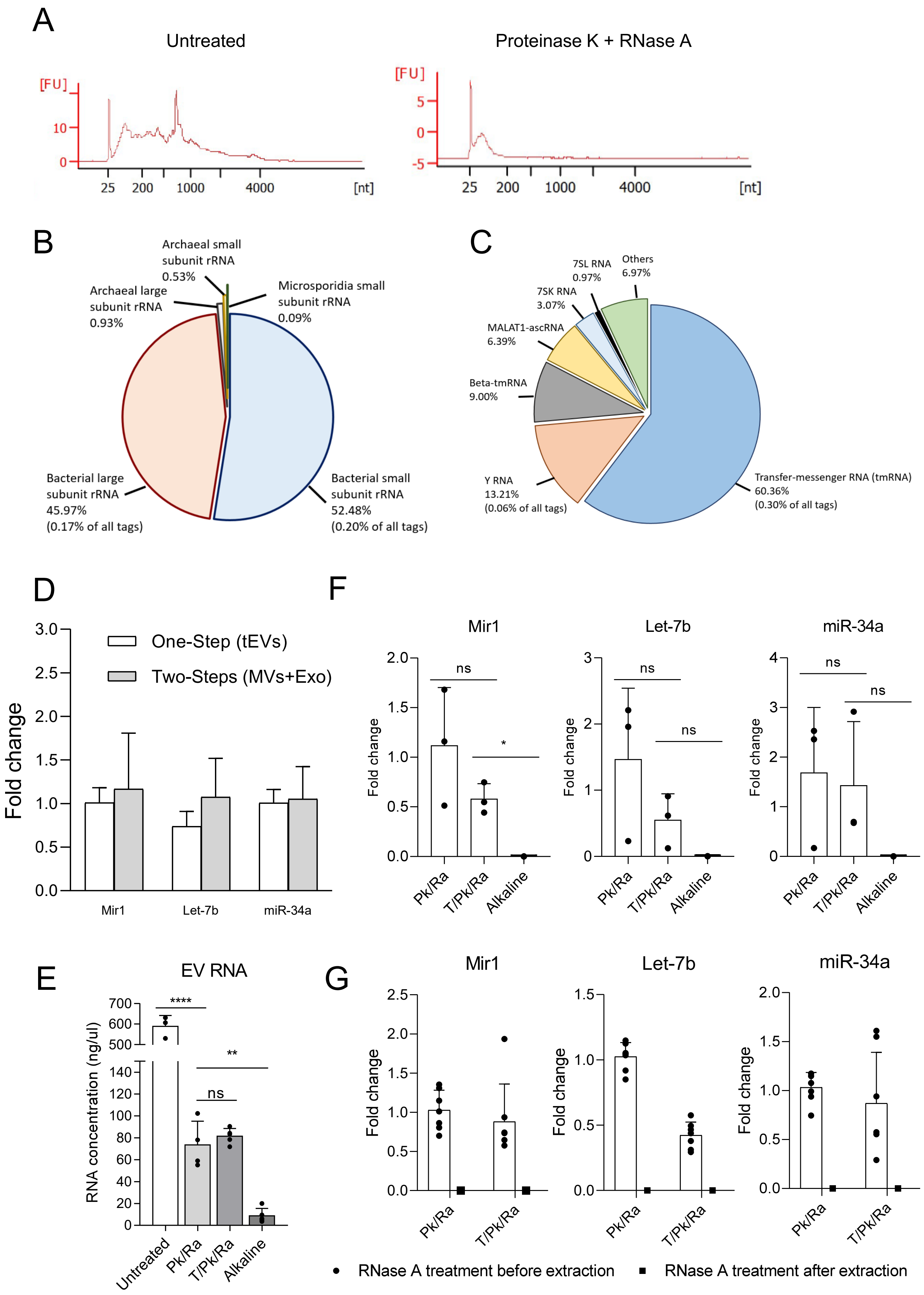

Additional file 1: Fig. S7| Characterization of the small RNA cargoes of testicular EVs.
