# Supplementary material for: The repertoire of testicular extracellular vesicle cargoes and their involvement in inter-compartmental communication associated with spermatogenesis
Source: BMC Biol. 2022 Mar 29;20:78. doi: 10.1186/s12915-022-01268-5 (PMC8966158; doi:10.1186/s12915-022-01268-5)
Supplement: Supplementary file 7 — Additional file 7: Figure S8. Uncropped blots. [file 12915_2022_1268_MOESM7_ESM.pdf]

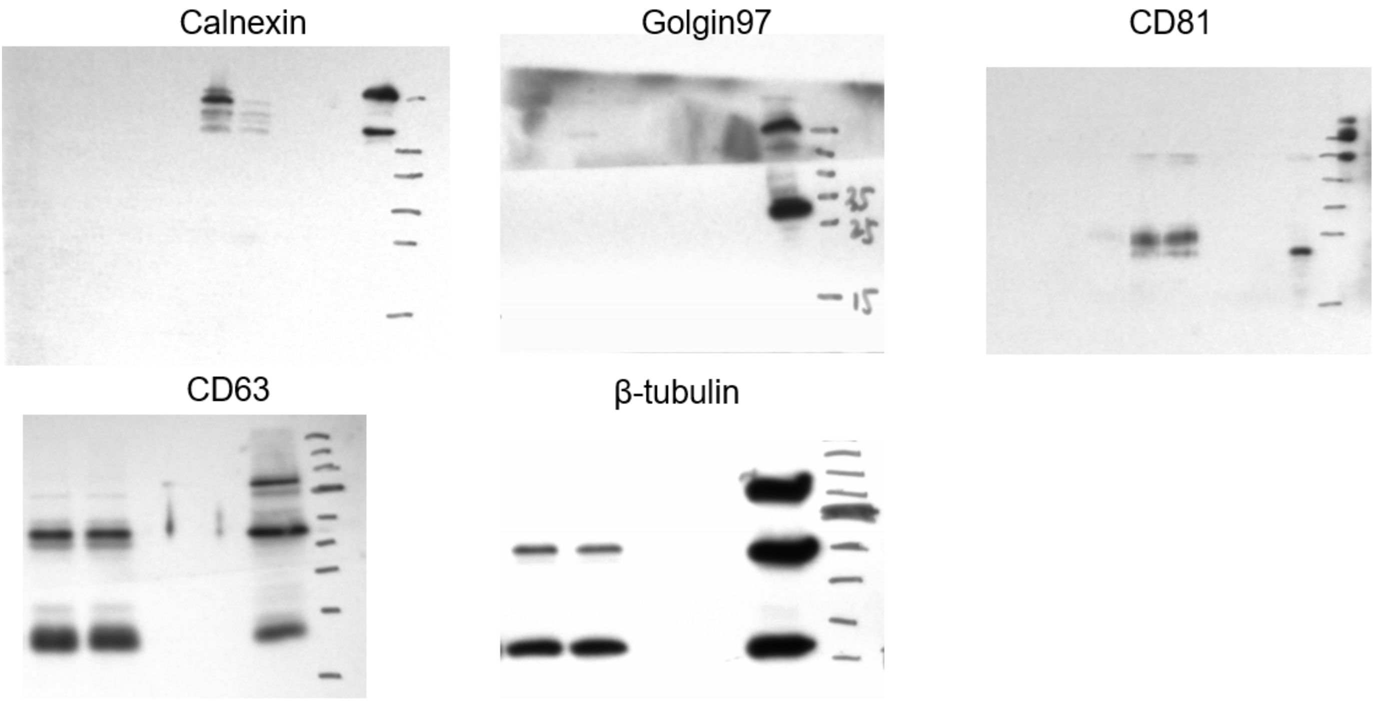

Fig 1| Characterization of testicular extracellular vesicles and their involvement in cell communication in the testicular microenvironment

BSG

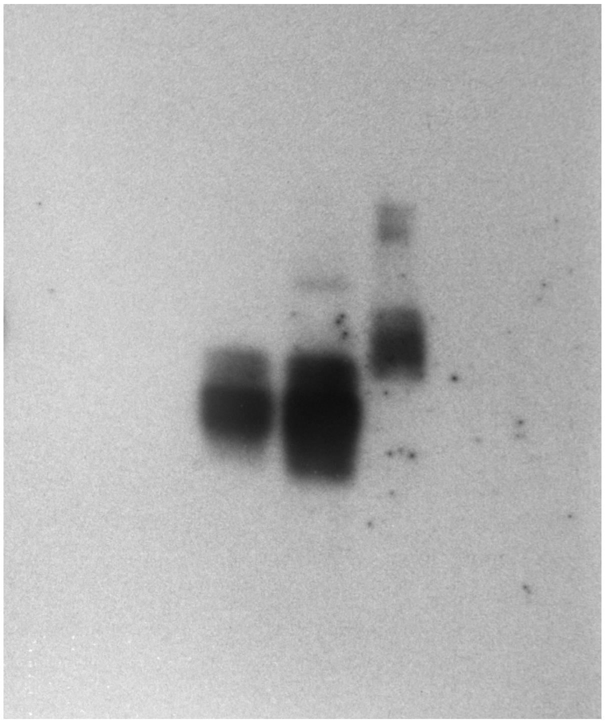

Fig. 2| Protein signature of testicular extracellular vesicles.

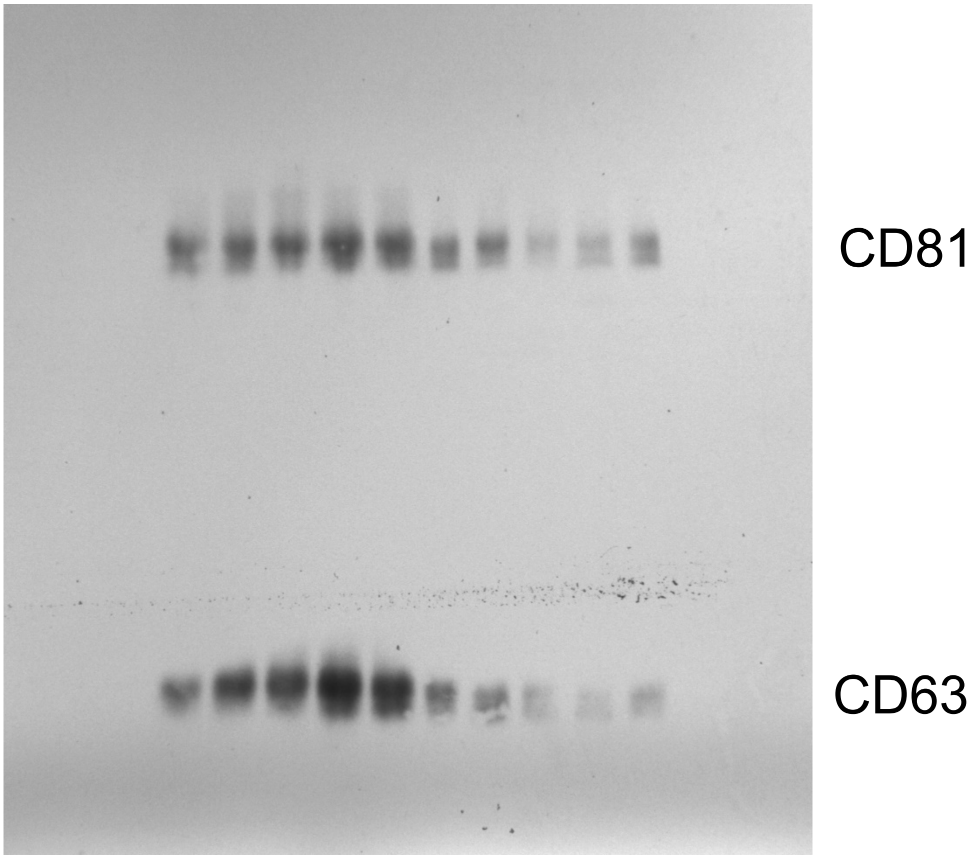

Fig 4| Inhibition of testicular extracellular vesicles elevated testicular germ cell apoptosis in mice

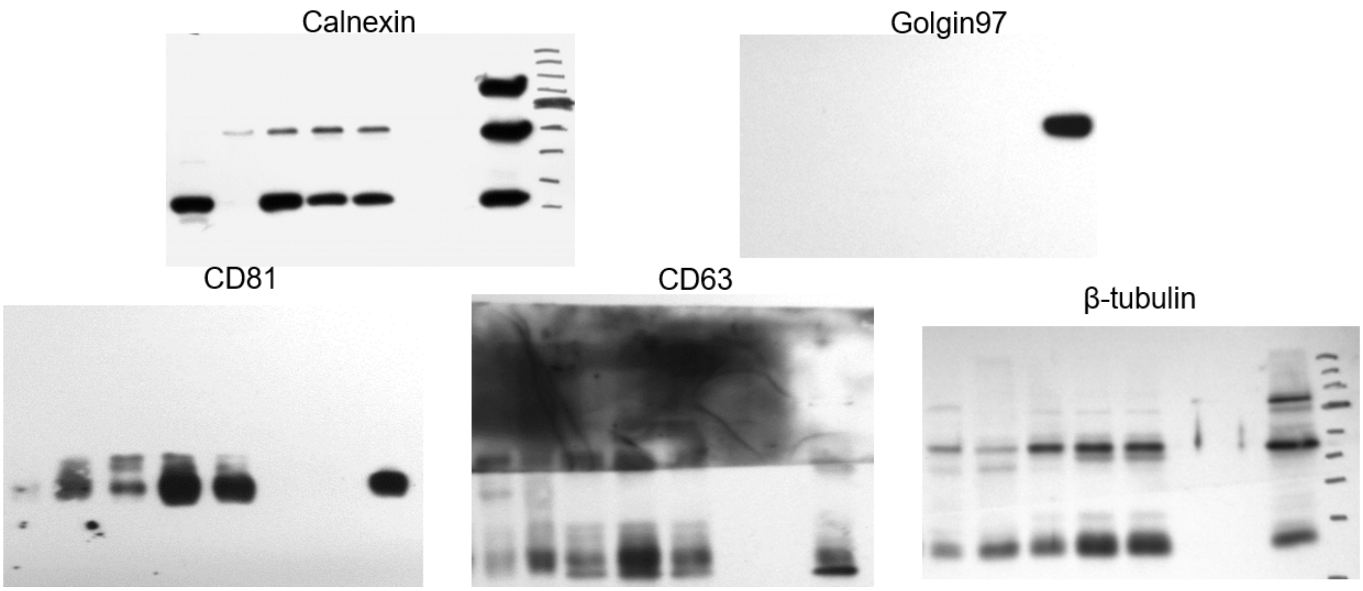

Suppl Fig S2| Isolation and characterization of extracellular vesicles in mouse testes and testicular cell lines
